# Investigating distortions in perceptual stability during different self-movements using virtual reality

**DOI:** 10.1177/03010066221116480

**Published:** 2022-08-09

**Authors:** Paul A. Warren, Graham Bell, Yu Li

**Affiliations:** Virtual Reality Research (VR2) Facility, Division of Neuroscience and Experimental Psychology, 12203 University of Manchester, Manchester, UK

**Keywords:** perceptual stability, perception and action, self-movement, virtual reality

## Abstract

Using immersive virtual reality (the HTC Vive Head Mounted Display), we measured both bias and sensitivity when making judgements about the scene stability of a target object during both active (self-propelled) and passive (experimenter-propelled) observer movements. This was repeated in the same group of 16 participants for three different observer-target movement conditions in which the instability of a target was yoked to the movement of the observer. We found that in all movement conditions that the target needed to move with (in the same direction) as the participant to be perceived as scene-stable. Consistent with the presence of additional available information (efference copy) about self-movement during active conditions, biases were smaller and sensitivities to instability were higher in these relative to passive conditions. However, the presence of efference copy was clearly not sufficient to completely eliminate the bias and we suggest that the presence of additional visual information about self-movement is also critical. We found some (albeit limited) evidence for correlation between appropriate metrics across different movement conditions. These results extend previous findings, providing evidence for consistency of biases across different movement types, suggestive of common processing underpinning perceptual stability judgements.

## Introduction

Perceiving a stable scene during self-movement is an impressive feat that is undertaken seemingly effortlessly by the human brain. To see the difficulty of this problem, note that any observer movement leads to a complex array of retinal motion such that the vast majority of world-stationary parts of the scene are actually moving on the retina. In the face of all this motion, how does the brain recover the percept of a largely stable environment? One important general solution—which we will refer to here as the *compare and cancel* solution—rests upon having access to multiple sources of information about self-movement and interpreting retinal motion in the context of that information. Under this solution, the brain effectively compares current retinal motion signals to information about self-movement provided either by predictive mechanisms that specify the visual input that should accompany that movement or by sensory feedback from other systems (e.g., vestibular or proprioceptive information). If the compared retinal and self-movement signals cancel each other out across the scene, then the scene is perceived as stable, whereas discrepancies would signal movement in/of the environment.

Perhaps the most prominent compare and cancel solution is associated with Helmholtz and his outflow theory for explaining why the world is perceived as stable when on observer makes an eye movement (Gregory, 1997). This was subsequently refined and extended in the reafference principle of von Holst and Mittelstaedt (1950) and von Holst (1954). In the terminology of von Holst (1954), the *afferent* sensory signal (i.e., the retinal input) can be considered the sum of the *exafferent* (incoming sensory signals due to movement in/of other parts of the scene) and *reafferent* (incoming sensory signals due to movement of the observer) components. Helmholtz's outflow theory suggests that the brain compares and cancels the afferent sensory signal (retinal input) with copies of the efferent motor commands sent to the eye muscles to bring about the movement. More recently, this account is commonly discussed in the context of forward models of motor control (Miall & Wolpert, 1996). In this framework, the motor command is the basis of a prediction (via a forward model) about the reafferent signal that should accompany the movement commanded. If the predicted reafferent (from the forward model) and the actual reafferent (from the retinal input) cancel each other out, then the world is perceived as stable. Of course, this theory is applicable not only to eye movements but motor commands for any type of observer movement.

While it is clear that motor command information is important for perceiving a stable environment during active, self-generated movement we do not lose the ability to perceive the scene as largely stable when engaged in passive movement (i.e., when not moving under our own steam). The world does not suddenly become instable when we are driving a car or moving on an escalator precisely because other sources of information about self-movement are available for comparison with the afferent retinal input. For example, vestibular information about movement is still available in such circumstances and can be used to aid in the process of interpreting scene relative movement during observer movement (MacNeilage et al., 2012). Of course vestibular input becomes unreliable if the observer is traveling at constant velocity. Also important then is direct visual information about self-movement, in the form of optic flow signals. Such information is always available to a moving observer (as long as the eyes are open and there are visible features in the visual scene) and a purely visual compare and cancel solution termed *optic flow parsing* has been the focus of extensive research in the last 15 years. Early work provided the first evidence for the existence of this mechanism (Matsumiya & Ando, 2009; Rushton & Warren, 2005; Rushton et al., 2007; Warren & Rushton, 2007, 2008, 2009a). Subsequent work has focused on its processing characteristics (Evans et al., 2020; Foulkes et al., 2013; Layton & Fajen, 2016; Rogers et al., 2017; Royden & Connors, 2010; Rushton et al., 2018a,b; Warren & Rushton, 2009b; Warren et al., 2012) and the interplay between flow parsing and non-visual systems providing self-movement information (Dokka et al., 2015; Fajen et al., 2013; Fajen & Matthis, 2013; MacNeilage et al., 2012; Niehorster & Li, 2017; Peltier et al., 2020).

Compare and cancel mechanisms have been studied extensively for recovery of head-centered speed during simple pursuit eye movements (e.g., Freeman & Banks, 1998) and scene-relative motion during simulated observer movement based on optic flow processing (e.g., Warren & Rushton, 2009a). However, more complex physical movements are much less well studied. This is, of course, understandable given the experimental difficulties encountered when trying to undertake such research. Pioneering work by Gogel (e.g., see Gogel, 1990 for summary) on perceptual stability during head movements used a simple point of light stimulus in an otherwise dark environment to show that a scene-stationary stimulus is sometimes perceived to move (i.e., perceptual stability is broken) when the observer moves the head. However, to study the limits of perceptual stability and the performance of the mechanisms that underpin this ability requires breaking the normal causal relationship between observer and scene movement on the retina—that is, the ability to independently move the stimulus during observer movement. This would enable use of a psychophysical approach to recover metrics that characterize the accuracy and precision of perceived stability of the scene during movement of the observer.

In order to manipulate the normal relationship between observer movement and retinal consequences of that movement, Wallach et al. (1974) used a highly innovative approach in which the movement of a physical stimulus was decoupled from (but still yoked to) that of a walking observer via a system of pulleys and gears. This study provided evidence that there are marked inaccuracies in perceptual stability during movement (although it did not use a psychophysical approach).

**Figure 1.**
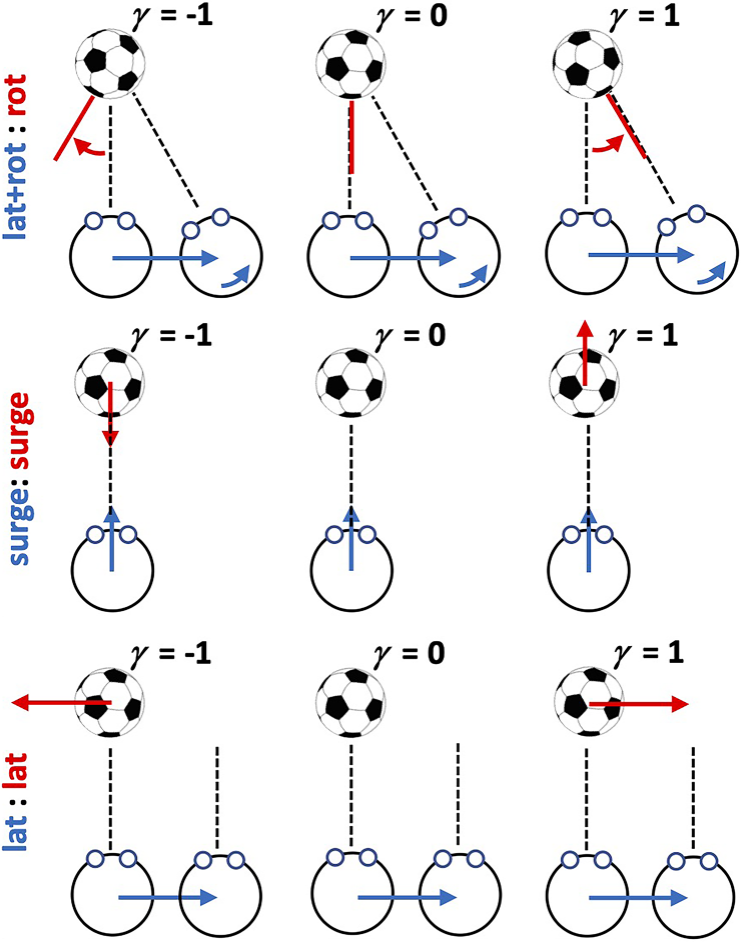
Schematic illustration of the three observer movement/target movement combinations.

More recently, the advent of head-tracking technology has enabled greater control over decoupling and yoking of stimulus and observer movement. Wexler (2003) used head tracking together with 3D stereoscopic shutter glasses to present of a 5 × 5 frontoparallel stimulus that could move in depth on a computer monitor. Crucially, via head tracking, the stimulus depth was updated in response to observer movement towards or away from the monitor. Movement of the stimulus was controlled via a gain parameter (see Figure 1, middle row) such that when gain was 0 the stimulus was stationary (at a fixed depth) in the scene. When gain was 1, the stimulus moved in the same direction and at the same speed as the observer (i.e., as the observer approached the screen the stimulus moved in the same direction as the observer and at the same speed). When gain was −1, the stimulus moved at the same speed as the observer but in the opposite direction. The participant's task was to indicate whether the stimulus was moving in the same direction as or in the opposite direction to their own movement. The experiment was repeated for both an active self-propelled moving participant (the voluntary condition) and a passive condition in which the participant was moved in a wheelchair by the experimenter (the involuntary condition). This study provided evidence for a significant bias in perceptual stability. More specifically, in order for the participant to perceive the stimulus as stationary (equally likely to report “same” or “opposite”), the gain had to be around 0.38 in the voluntary condition and 0.57 in the involuntary condition. This means that to perceive the stimulus as stable in the scene, it actually had to move markedly in the same direction as the participant at 38% (voluntary) and 57% (involuntary) the speed of observer movement. Note that this result suggests that the bias was larger in the involuntary condition, that is, when there was less information about self-movement, since the motor command information (and potentially proprioceptive information) was no longer informative. Similarly, this study showed that sensitivity to the gain manipulation was higher in the voluntary condition. Taken together these data are consistent with the idea that voluntary movement yields better performance but reveal a perhaps surprising level of inaccuracy in the systems that perceive (in)stability in the scene.

Using a similar psychophysical method to Wexler (2003) but now adapted to employ immersive Virtual Reality (VR) technology, Tcheang et al. (2005) presented an elegant VR-based extension of Wallach et al. (1974). Specifically, the target stimulus was a virtual football at eye height which could rotate about its center. Participants made lateral translation movements past the football which, depending on the gain parameter (similarly defined to that in Wexler, 2003), either turned with the observer or against the observer at a variable proportion of observer speed (see Figure 1, top row). When gain was 0, the ball was scene stationary. When gain was 1, the ball turned to present the same aspect at the observer moving past it. When gain was −1, the ball rotated away from the observer at the same angular speed. The results were similar to those of Wexler (2003) in that for the ball to be perceived as stationary it had to rotate with the observer with a gain between 0.2 and 0.45 over the 4 participants tested. Tcheang et al. (2005) also provided evidence for the importance of other sources of information about self-movement for perceiving s stable scene. The bias markedly reduced and the sensitivity to the gain manipulation (i.e., to scene instability) increased significantly when there was a background which provided rich optic flow information (i.e., visual information about self-movement).

In the present study, we use a similar psychophysical approach to that of Wexler (2003) and Tcheang et al. (2005) to investigate perceptual stability in VR across a range of movement conditions. We use the same basic manipulation of a gain parameter to alter the relationship between observer movement and the yoked movement of a target object about which the participant makes judgements. As noted above, Wexler (2003) considered only forward/backward translations of the observer and accompanying forward/backward translations of the target (with 11 participants). In contrast Tcheang et al. (2005) considered only lateral translation of the observer and counter rotation of the target (in 4 participants). Here we will consider both these observer-target movement combinations (OTMCs) as well as a third combination in which the participant and target both translate laterally. Moreover, we will recover bias and sensitivity parameters to scene relative target movement in the three conditions across the same set of 16 participants. In particular we will examine whether there is evidence for similarities and/or relationships between these parameters across conditions which would be suggestive of common underlying processing for different OTMCs. In addition, similar to Wexler (2003), we will also consider the important question of how the parameters change when participant movement is active (i.e., participant generated) versus passive (experimenter generated), a manipulation which affects the amount of information about observer movement available to the brain. We anticipate that bias should be lower and sensitivity higher when the observer is actively moving. The extent to which these parameters change will then reflect the additional contribution of motor command information about self-movement (efference copy) on perceptual stability.

## General Methods

### Design

We manipulated two experimental factors using a full factorial within participants design across six conditions. The first factor was OTMC with three levels. For clarity, we use the notation “observer movement:target movement” to label factor levels. We use the term “Lateral” (L) to refer to leftward/rightward translational movements, “Surge” (S) to refer to forward/backward translational movements, and “Rotation” (R) to refer to (yaw) rotations. The three factor levels investigated were: (a) (Lateral + Rotation):Rotation (L + R):L for short, which is similar to the observer-target movement investigated in Tcheang et al. (2005); (b) Surge:Surge or S:S for short which is similar to the observer-target movement investigated in Wexler (2003); and (c) Lateral:Lateral or L:L for short. Figure 1 provides an illustration to explain each of these 3 factor levels. For the (L + R):L conditions participants made a lateral movements while simultaneously counter rotating the head to remain looking at the center of the target which could independently rotate about its center. For the S:S condition, participants made forwards/backwards movements while maintaining looking straight ahead at the target which also translated forwards and backwards. For the L:L condition, participants made lateral movements while maintaining looking straight ahead while the target also translated laterally. The second factor was the observer movement generation type (MGT) with two levels: active and passive. In active conditions, participants moved in a controlled manner under their own steam (see procedure below), whereas in the passive conditions they were moved in a similar manner but on a custom-made linear track by the experimenters (see procedure below). The dependent variables were the location and slope parameters of the psychometric function fitted to response data (see below).

Conditions were blocked by the MGT factor such that participants did either all passive or all active conditions first (counterbalanced across participants). The order of the three OTMC levels was randomized for each participant within each of the passive or active blocks. Data for each MGT block were typically collected on separate days with each session lasting around 75 min.

### Participants

Sixteen participants (all students at the University of Manchester, two were non-naïve and involved in data collection) were recruited. Ethical approval for the study was provided by the University of Manchester Research Ethics Committee, and informed consent was given by all participants before beginning the experiment.

For each condition, that is, for each OTMC (3 levels) × MGT (2 levels) combination, our aim was that each naïve participant would undertake 3 six-minute runs. Consequently, a complete data set for naïve each participant would comprise 18 runs in total. However, in practice, we did not achieve this aim with all participants. Of our 14 naive participants, 12 completed the full data collection schedule. P09 provided the desired three repeated runs in all conditions except (L + R:R, Passive) for which no runs were completed. P09 provided the desired three repeated runs in all conditions except (L + R:R, Passive) for which only 2 runs were completed. We also added the data from the two non-naïve participants (P01 and P02). P01 provided three repeated runs for the (S:S, Passive) but only two repeated runs for all other conditions. P02 provided three repeated runs for the (L + R:R, Active) condition and two repeated runs for all other conditions except (L + R:R, Passive) and (L:L, Passive), which were not completed. Based on all these available data, we were able to recover psychometric function parameters from *N* = 16 participants for all conditions except (L + R:R, Passive) (*N* = 14) and (L:L, Passive) (*N* = 15).

### Stimuli

Stimuli (and experiments) were coded using Vizard (WorldViz, Santa Barbara, USA), a python-based VR development platform. The basic stimulus was similar to that used in Tcheang et al. (2005), that is, a typically sized (diameter ∼22cm) virtual football (soccer ball) presented at an initial distance of 1.5 m from the participant. The movement of the stimulus was yoked to that of the participant via a gain parameter *γ*, which could vary between −1 and 1 in each of the three levels of the OTMC factor as described in Figure 1.

### Apparatus

Data were collected at the Virtual Reality Research (VR2) Facility at the University of Manchester. We used the HTC Vive Head Mounted Display (HMD) linked to a wearable backpack PC (Zotac VR Go, ZBOX-VR7N70 16 GB Ram, 1070 GPU I7 6700T CPU). HMD resolution was 1,080 × 1,200 pixel in each eye at 90 Hz. Using this approach, participants could move freely and safely without being tethered. A previous investigation of the tracking performance of this system (Niehorster et al., 2017) has reported that it is precise and low latency but there is evidence for some systematic bias in roll, pitch, and eye height measurements. However, these issues should not have a large impact on our data since our participant movements are: (a) small in magnitude compared with the tracking region tested by Niehorster et al. (2017) and (b) restricted to a central region where observed biases were smallest.

Participants made responses on each trial by pressing one of two buttons on a handheld controller. In order to make observer movements as regular as possible, a table (1.5 × 0.45 m) was placed either in front of (lateral observer movement conditions) or next to (forward/backward observer movement conditions) the participant. We also used a freely available metronome app, which presented an audio stimulus at 40 beats per minute (bpm) in order to better control observer movement.

**Figure 2.**
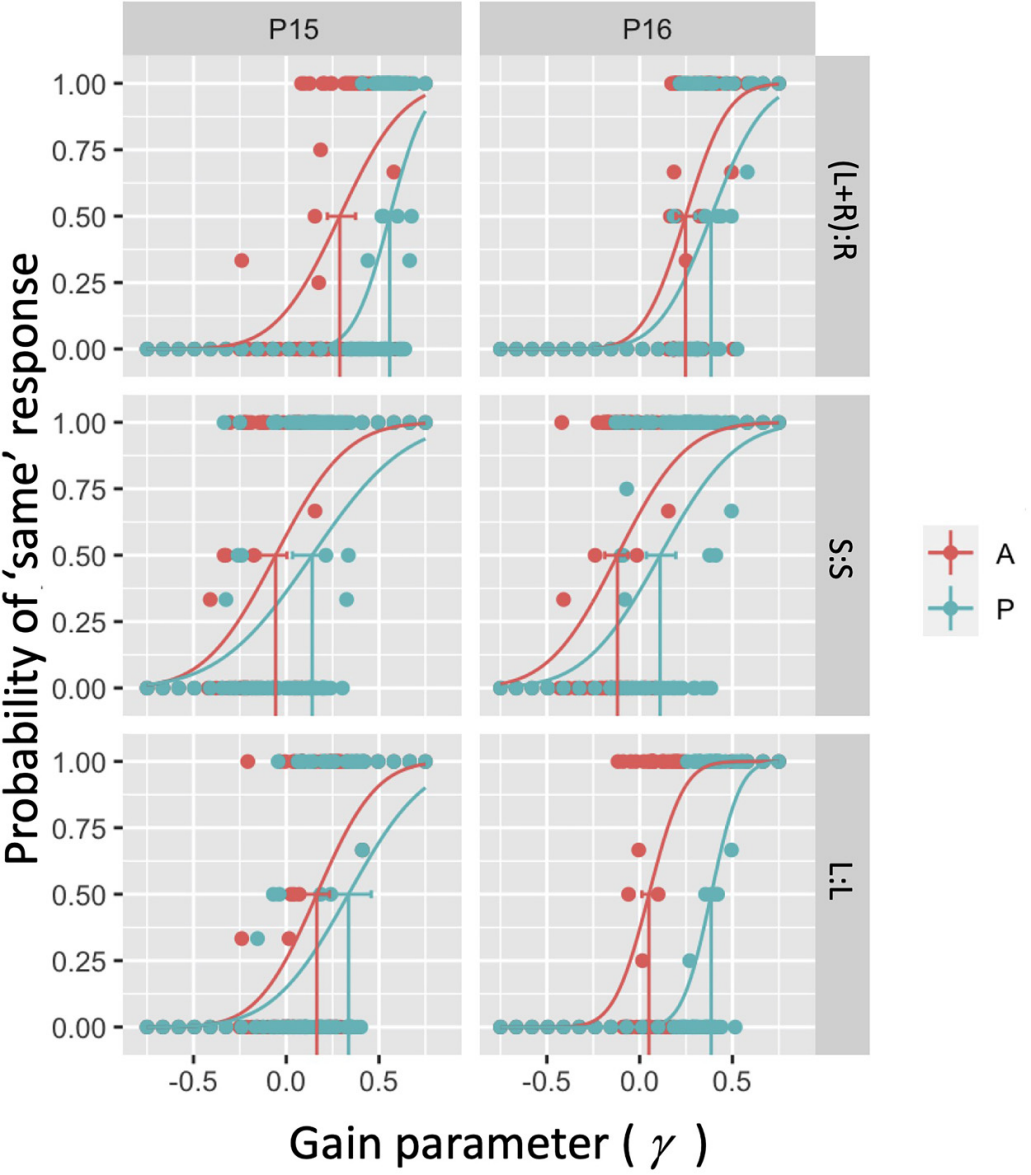
Example psychometric functions for two (P15 and P16) of our 16 participants in the six conditions considered. In each panel, the dots correspond to local average estimates over the binary response variable. Curves correspond to fitted psychometric functions to 120 underlying binary responses using a cumulative Gaussian psychometric function form.

In the passive movement conditions, a rotating chair was securely fastened to a 120 cm diameter circular platform on a set of 4 fixed trolley wheels (see Figure 2). The chair could be rotated on the platform so the shoulders of someone sitting in the chair were either parallel (for lateral movement) or perpendicular (for forward/backward movement) to the wheel direction. Placing the trolley wheels in a 200 × 80 cm metal track enabled smooth movement of the chair in the direction of the wheels. By rotating the chair and the track in different ways and with two experimenters pushing on either side of the chair, we could control smooth passive lateral or surge observer movements.

### Procedure

After putting on the HMD participants first acclimatized to a virtual environment in the HTC home space. During this period, we monitored participants for signs of nausea (although no such effects were reported by participants). After the acclimatization period had ended participants either sat in the chair (passive conditions) or stood against the table (active conditions). In both cases, the participant's initial position was aligned as closely as possible with an origin point relative to which position was measured. In order to make this point as consistent as possible across participants, a guardian was created within Steam VR and the center point of this play space was visible. This gave a visual guide for where the participant should start from in the virtual space. In practice, this position was very similar across participants and the position was marked with tape on the floor. Once the participant was stood in the correct position, they were asked to look straight ahead and the experiment began with the ball appearing directly in front of them.

In the active conditions on each trial, participants were instructed to make lateral (left/right) or surge (forwards/back) movement while either looking straight ahead (L:L) or maintaining fixation at the center of the ball (L + R:R). For lateral observer movement conditions, participants were instructed to stand with feet at shoulder width apart and shift weight between their two feet to make movements of around 35–40 cm (approximately one shoulder width) left and right repeatedly. For the L + R:R condition, the accompanying maximum counter-rotation of the head required to maintain fixation on the football was around 6–7 degrees from straight ahead. For surge observer movement conditions, participants were asked to stand with one foot in front of the other (separated by around shoulder width) and made backwards and forwards movements repeatedly from the back foot to the front foot. In both condition types, participants were asked to make movements as close to linear as possible using the table as a guide and changing direction in time with the metronome clicking at 40 bpm. Note that in line with Wexler (2003) and Tcheang et al. (2005) the movements were constrained but we did not try to perfectly match movements across participants; indeed, the movements were described in terms of the naturally varying shoulder width of each participant. However, the gain parameter (as described above) is unit free, and irrespective of the exact extent of the movement, it is consistent across participants.

In passive conditions, participant movement was controlled by two experimenters pushing on the arms of the chair in the direction (lateral or surge) of the linear track and changing direction in time with the metronome until a response was made. In the L + R:R condition, experimenters controlled the lateral movement of the observer and we assume that the dominant source of information about how much the ball should rotate in response to the observer movement comes from this lateral displacement. Consequently, in the passive condition, participants were still instructed to make the (small) head rotations undertaken in the active condition to maintain gaze direction at the center of the ball. Markers were placed on the floor to match approximately the extent of the lateral and surge movements so that passive movement extent and speed were as closely matched as possible in active and passive conditions.

On each trial, the movement of the ball was yoked to the movement of the observer via the gain parameter *γ* (see Figure 1 and text above). The participant's task was to indicate in a 2-Alternative Forced Choice (2AFC) paradigm whether the football moved either in the same direction as or opposite direction to the observer movement. The gain parameter was altered on each trial via an adaptive Kesten staircase procedure (Treutwein, 1995). If the participant perceived the ball to rotate with them then *γ* was decreased and if they perceived the ball to rotate in the opposite direction then *γ* was increased. In each short data collection run, which incorporated a single experimental condition and lasted around 6 min, we interleaved two such Kesten staircases, each containing 20 trials (i.e., 40 trials in total per run). Note that this staircase is particularly quick to converge and in practice 20 trials for each staircase was sufficient. One staircase started from a trial at *γ*  = 0.75 (i.e., clearly leading to a “same” response) and was designed to converge to a point at which the participant made 15% “same” responses. The other staircase started from a trial at *γ*  = −0.75 (i.e., clearly leading to an “opposite” response) and was designed to converge to a point at which the participant made 85% “same” responses. This choice of crossing staircases ensured that there was most data in the region where responses were most uncertain—allowing more robust psychometric function fitting and parameter estimation.

### Psychometric Function Fits

For each of the six conditions, we modeled 2AFC responses using a cumulative Gaussian psychometric function relating the gain parameter to the probability of responding “same.” Each psychometric function was fitted to data from 120 trials for the majority of participants and conditions (and 80 for the others). Fits were conducted using the quickpsy library (Linares & López-Moliner, 2016) in RStudio (RStudio Team, 2021). From the fitted psychometric functions, we recovered the gain value at which participants were equally likely to say “same” or “opposite.” Under an assumption that a symmetric psychometric function is appropriate to model this data (which does not appear unreasonable), this is the point of subjective stationarity (PSS), that is, the point at which participants perceived the object to be scene stationary during self-movement. Any departure in the PSS from a gain of zero suggests bias in the percept (i.e., the football needed to move in the scene to be perceived as scene stationary). We also recovered the standard deviation of the underlying fitted Gaussian which reflects sensitivity to the gain manipulation (i.e., a measure of how much tolerance is present when stability is perturbed).

Note that because a small number of participants did not complete all conditions, there were missing values in three out of 96 (16 participants × 6 conditions) cells for the PSS data and the corresponding cells for the Gaussian s.d. data.

## Results and Interpretation

### PSS Parameters (Bias)

Figure 4A illustrates the PSS (i.e., the bias) in observer settings for the six conditions tested. Note that biases are predominantly positive across the three OTMCs and also irrespective of whether movement is passive or active. The lower extremes of the 95% confidence interval for PSS values in all 6 conditions were all above 0 (see Table S1 in supplementary materials). Biases are in the same direction as observed in previous experiments and appear similar in magnitude on average to the equivalent of our (L + R):R condition (Tcheang et al., 2005) and S:S condition Wexler (2003). Note also that biases appear higher in the passive relative to active conditions.

To model our data, we fit and compare a set of five nested linear mixed effects models. All five models incorporate a random effect of participant but differ in the presence of fixed effects associated with the two independent variables, MGT and OTMC. We compare fits based on AIC values and nested likelihood ratio tests (LRTs—e.g., see Mood et al., 1974). Model 1 is the null model, which incorporates just the random effect of observer (intercept only). All subsequent models also incorporate observer as a random intercept effect. Model 2 (OTMC) and Model 3 (MGT) incorporate only one of the independent variable predictors in isolation. Model 4 (OTMC + MGT) includes both independent variable predictors but no interaction. Model 5 includes both independent variable predictors and their interaction (OTM*MGT = OTMC + MGT + OTMC × MGT). Results of this analysis are presented in Table 1. The calculated AIC values associated with the fits are presented in the first 5 rows of the table (second column) together with the AIC weights (third column). Table 1 suggests that the model involving only MGT was the best fitting model taking number of parameters into account. This was also confirmed via nested hypothesis testing using a series of LRTs to compare fits of nested models.

**Table 1.** AIC values and AIC weights for five linear mixed effects models fitted to both PSS and Gaussian s.d. data. Highlighted cells correspond to lowest AIC values (and thus best fitting models). In the second part of the table, we present the outcome of likelihood ratio tests for model comparison, confirming that Models 3 and 4 were best for PSS and s.d. parameters, respectively.

Model	PSS: AIC	PSS: AIC weight	s.d.: AIC	s.d.: AIC weight
**1. NULL**	***−31.92***	***0.020***	***−69.64***	***0.043***
**2. OTMC**	***−29.53***	***0.006***	***−72.15***	***0.150***
**3. MGT**	***−39.07***	***0.699***	***−71.79***	***0.125***
**4. MGT + OTMC**	***−36.92***	***0.239***	***−74.88***	***0.588***
5. MGT*OTMC	***−33.18***	***0.037***	***−71.20***	***0.094***
Model comparison		LRT^1^	df^2^	*p*-value
PSS: 2 vs. 1		*1.61*	*2*	*.448*
**PSS: 3 vs. 1**		*9.15*	*1*	.002**
**PSS: 4 vs. 3**		*1.85*	*2*	*.396*
**PSS: 5 vs. 3**		*2.11*	*4*	*.716*
**SD: 2 vs. 1**		*6.51*	*2*	.039*
**SD: 3 vs. 1**		*4.15*	*1*	.042*
**SD: 4 vs. 2**		*4.73*	*1*	.030*
**SD: 4 vs. 3**		*7.0881*	*2*	.029*
**SD: 5 vs. 4**		*0.3296*	*2*	*.848*

*Note*. AIC = akaike information criterion; df = difference in degrees of freedom for compared models (df for the chi-square test); LRT = the likelihood-ratio test statistic (chi-square); PSS = point of subjective stationarity; s.d. = standard deviation.

Specifically we show that model 2 (OTMC) is not better than model 1 (null model), whereas model 3 (MGT) is better than model 1 (null model). However, neither of model 4 (OTMC + MGT) or model 5 (OTMC*MGT) is better than model 3. This analysis suggests that the MGT factor is the primary driver of differences in PSS and that the OTMC factor has limited impact on PSS.

Taken together, these analyses suggest that, as anticipated, and in line with previous research, biases were positive (cf., Tcheang et al., 2005; Wexler, 2003) and elevated in active relative to passive conditions (cf., Wexler, 2003). Interestingly, this seems to be the case irrespective of what kind of compensation for target movement is required during self-movement.

### Gaussian S.D. Parameters (Sensitivity)

**Figure 3.**
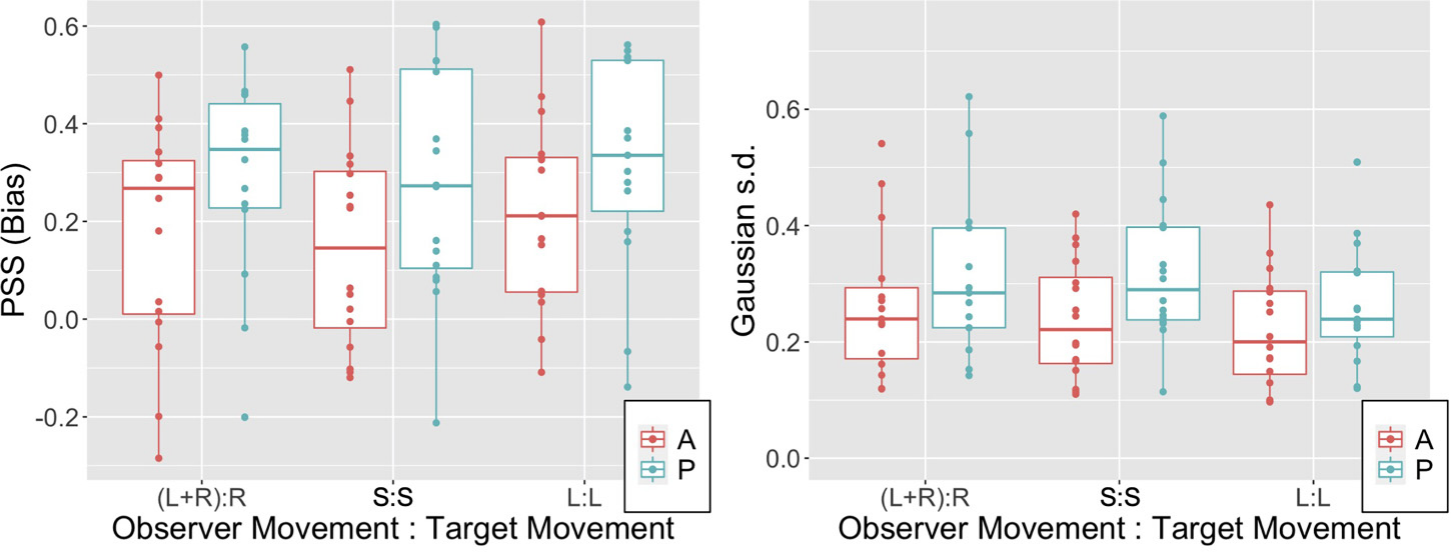
PSS (left panel) and Gaussian s.d. (right panel) data for the three OTMCs and both active (A) and passive (P) movement generation types. Boxplots illustrate median (thick horizontal line), 25^th^ and 75^th^ percentiles (hinges) and data points less than 1.5 × IQR from the hinge (whiskers).

Figure 3B illustrates the Gaussian s.d. (i.e., reciprocal of sensitivity) of the fitted psychometric function for the six conditions tested (note 2 extreme points, both above 0.85 and both occurring in the (L + R):R conditions are not shown). Table S2 in supplementary materials reports the 95% confidence intervals for this parameter.

Similar to the PSS parameter note that again the fitted Gaussian s.d. appears to be higher (i.e., sensitivity appears to be lower) for Passive relative to Active conditions across all OTMCs. To model the Gaussian s.d. data, we again fit the 5 linear mixed effects models outlined above. The calculated AIC values associated with the fits are presented in the first 5 rows of the table (third column) together with the AIC weights (fourth column).

Model 4 involving the sum of the OTMC and MGT factors has the lowest AIC value and thus provides the best fit taking number of parameters into account. This is also confirmed via nested hypothesis testing using a series of LRTs to compare fits of nested models. This analysis showed that Models 2 (OTMC) and 3 (MGT) are both better than the null model but Model 4 (OTMC + MGT) is better than both models 2 and 3 and Model 5 (OTMC*MGT) is not better than model 4.

This analysis suggests that both the MGT and OTMC factors drive differences in the Gaussian s.d. parameter and that, in line with Wexler (2003), sensitivity to scene relative target movement during self-movement is increased in active relative to passive conditions. However, unlike the PSS parameter, there is now some evidence that different combinations of observer-target movement lead to differences in sensitivity. Subsequent comparison of the Gaussian s.d. parameters across the three OTMC levels (averaging over the MGT levels and using Tukey's HSD) provide evidence for a difference only between the L + R:R and L:L conditions (*t*(80.4) = 2.647, *p* = .026).

### Correlation across OTMCs

In the analyses of accuracy and precision parameters outlined above, we found mixed evidence for whether the OTMC was important. If compensation for self movement in order to perceive a stable scene rests upon similar processing irrespective of the OTMC requiring compensation, then we might hypothesize the existence of correlations between our parameters across the same participants in different OTMC conditions. To provide a preliminary examination of this hypothesis, we calculated three Pearson's *r* values (one for each distinct pair of the three OTMC levels) for both Passive and Active data separately and for both the PSS and Gaussian s.d. parameters. Applying a Bonferroni correction (*α* = 0.05/6 = 0.008) for the six tests conducted on each parameter type, we found that there were no significant correlations for the PSS parameter. However, for the Gaussian s.d. parameter, we found significant positive correlations over participants between S:S and L:L during active movement (*r*(14) = 0.6513, *p* < .005) and between (L + R):R and L:L during passive movement (*r*(14) = 0.6638, *p* < .005).

## Discussion

### Summary

Across three different OTMCs we measured both accuracy and precision of judgements about the scene stability of an isolated target during both active (self-controlled) and passive (experimenter-controlled) observer movement. In line with previous research (Tcheang et al., 2005; Wexler, 2003), we found evidence for a marked positive bias such that the target had to move with (in the same direction as) the observer in order to be perceived as scene stable. We also found that bias increased and sensitivity to scene instability decreased when observers made passive relative to active movements (cf., Wexler, 2003). We have extended this research, showing that:

1. Bias appears to be consistent in direction and magnitude across a range of OTMCs.
2. The increase in bias and decrease in sensitivity observed previously in passive relative to active movements is present across all different OTMCs tested. However, large biases are still present in even when efference copy information is available.
3. There is some limited evidence for correlations between commensurate measures across different OTMCs, suggesting potential common processing to support perception of scene stability across movement types. However, the apparent impact of movement type on sensitivity to scene instability is inconsistent with this suggestion.

### Large Biases in Perceptual Stability

The biases reported here and in previous studies are perhaps surprisingly large. For example, in the L:L condition, our data suggest that when making a lateral movement at an average speed of around 26 cm/s, the participant would require a movement at around 25% of this average (6.5 cm/s) in the same direction as the observer in order to perceive the target as scene stable. However, note that this experiment was conducted in a very sparse scene containing only the target. In natural viewing, there are other objects against which relative movement can be compared and there is additional (purely visual) information available about self-movement from optic flow. When such additional information is presented in the scene, the biases are largely eliminated (see Tcheang et al., 2005). In particular, when the target was presented with a wireframe background at a different depth in the environment from which a rich optic flow field was available the bias was halved. This result suggests the importance of purely visual information for perceiving a stable scene and is consistent with the extensive work on optic flow parsing cited in the introduction (e.g., Warren & Rushton, 2009a). It is interesting, nonetheless, that there is such a high level of inaccuracy in the sparse environment used in our experiment. This finding together with the marked reduction in bias that occurs when information about self-movement is available from efference copy (in our active conditions) speaks to the importance of the presence of multiple information sources about self-movement. When any one of these is missing, there is potential for considerably larger bias.

### Comparing Passive and Active Movement

By comparing changes in bias and sensitivity in the active versus passive conditions, we can estimate the contribution of efference copy information about self-movement (which is present in active but not passive conditions). Based on our data, we suggest that the presence of efference copy information leads to an average reduction in bias of around 39% (comparable to an estimate of 36% based on Figure 2 in Wexler, 2003) and a reduction in Gaussian s.d. of around 21% (comparable to an estimate of 25% based on Figure 2 in Wexler, 2003). This suggests that efference copy information plays a significant role in our ability to accurately and precisely perceive a stable scene but is by no means sufficient (biases are still significant in the active condition). We suggest that visual information about self-movement available from optic flow is also important for perception of stability, and the fact that this is absent is a reason for the persistence of bias even when efference copy is available (see also data from Tcheang et al., 2005). This finding is in line with research on optic flow parsing which suggests that a primary reason for our sensitivity to optic flow is that it allows us to interpret scene-relative movement during self-movement (e.g., see Warren & Rushton, 2009a). In future research, we will consider the relative contributions of visual, efference copy, and other information sources about self-movement to perceiving a stable scene.

### Common Processing to Support Perceptual Stability Across Different Movement Types

Statistical modeling revealed that the OTMC did not impact upon biases observed but did impact upon sensitivities to scene instability. However, inspection of Figure 3 suggests that such effects are rather subtle. We also found some evidence for correlation between sensitivity parameters across different OTMCs although we note that our sample size was perhaps too small to make strong claims based on correlation. Taken together, we suggest that there is some weak evidence for common processing of perceptual stability judgements across the conditions tested—or at least we cannot rule out the idea that common processing supports perceptual stability judgements across conditions. It is interesting to note that all our conditions involved some translation of the observer and so, if correct, then this might underpin this result. Observer translation is typically accompanied by a rich set of cues that provide strong information about 3D location and movement (e.g., visual parallax). It is possible that common associated mechanisms drive the similarity of effects observed across movements here. In future, it would be useful to consider whether such evidence persists when pure rotational versus translational observer movements are compared.

### Potential Causes of Bias

As noted above, it is likely that a most significant reason for bias in our data is that the available self-movement information is limited. However, the question remains of why the bias is positive. As noted by Tcheang et al. (2005), the positive bias observed in the PSS is consistent with one of two explanations—either an underestimate of the observer's percept of self-movement distance or an overestimate of the perceived distance from the observer to the target. However, the Tcheang et al. (2005) data were more consistent with the hypothesis that participants underestimated the distance they had moved. More specifically, when the experiment was repeated at different viewing distances, the recovered estimate of mis-perceived distance required to explain the data was inconsistent with considerable previous research. In particular, the perceived depth required was an overestimate and increased with viewing distance—the opposite effect to that reported previously (e.g., Johnston, 1991; Ogle, 1950). Moreover, there is considerable evidence that walked distance traveled is underestimated both in VR and in real life (e.g., see Durgin et al., 2005; Philbeck et al., 2004). Although our participants didn’t actually walk anywhere (they simply rocked side to side or back and forwards) it is possible that a similar tendency to underestimate distance moved might play a role here.

**Figure 4.**
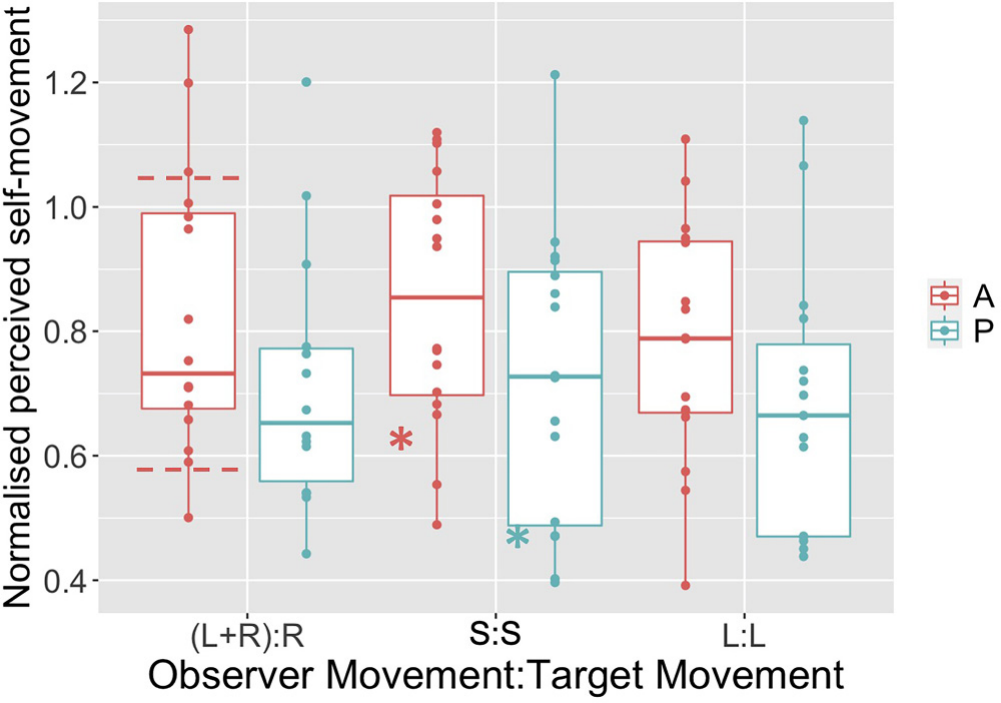
Normalized perceived self-movement distance that would explain the bias observed in our six conditions. Horizontal dashed lines on the Active, (L + R):R condition represent approximate range of equivalent data from Tcheang et al. (2005). The two stars on the S:S condition data represent approximate mean values for the equivalent conditions from Wexler (2003).

It can be shown (see supplementary material) that the equivalent normalized underestimate of distance traveled (i.e., the ratio of the perceived and actual distance traveled) that would explain the bias is given simply by 1-*β*, where *β* is the bias (i.e., the recovered PSS). Based on this calculation, in Figure 4 we present the normalized estimates of perceived distance traveled required to explain the bias. Note that our data are broadly in line with previous data, particularly that from Figure 9b in Tcheang et al. (2005), which were also collected using VR (horizontal dashed lines in Figure 4). However, equivalent average values from Figure 2 in Wexler (2003) are smaller than ours (the stars in Figure 4). This may be because that experiment used a markedly different apparatus—stimuli presented stereoscopically using stereo shutter glasses on a standard computer monitor.

### Potential Limitations

As with any study of this kind, in which the aim is to investigate aspects of perception with genuinely active observers, there are potential limitations. The first issue relates to the fact that it is impossible to constrain participant activity to the extent that they all make the same movements and completely follow the instructions. We did try to constrain movements by (a) providing a guide (the table) against which to move (b) providing the metronome which at least made pace approximately consistent. We primarily followed the methods of Wexler (2003) and Tcheang et al. (2005) who also did not constrain the movements of their participants beyond these methods. While it was never our intention to match movements between observers perfectly (indeed we explicitly asked them to move the extent of their own shoulder width), one might argue that by not constraining movement further we added additional noise to measurements taken. We acknowledge that this is possible but suggest that this would only make judgements less precise (making it harder to find any effects of our manipulations) as opposed to generating the clear biases observed. Note also that ball movements were yoked to the movement of the observer irrespective of distance traveled, and the gain parameter was unit free. So irrespective of exactly how far observers moved, the metric is still well-defined and consistent across participants as a measure of the extent to which the ball “followed” them. So, for example, a gain value of 1 still meant that the ball moved as if to completely counteract the effect of the observer movement irrespective of exactly how far that movement was.

A second issue concerns the difficulties associated with trying to match the movements in the active and passive conditions—to do this exactly would require us to (a) record the movement and (b) use a sophisticated system that could recreate that movement, for example, a motion platform. Instead we used similar (although arguably better constrained) methods to those of Wexler (2003) such that the experimenter simply moved the participant along a linear track in time with the metronome and over an extent that matched the average displacement of participants. If we had done this perfectly then, due to the information lost in the passive case, we would expect to observe precisely the pattern of results obtained (i.e., increased bias and an increase in the Gaussian s.d. parameter for passive movements relative to active movements). The fact that we were indeed able to observe this effect suggests that our approach was sufficiently good.

A third issue concerns the possibility of unwanted eye movements and/or head movements. It was not possible to record eye movements within our HMD experiment and we could not be sure that participants were perfectly following instructions with respect to where to direct gaze. Similarly (as noted above), we could not be sure that participants made head movements as instructed. While participants did not express any difficulty carrying out the task, such movements would lead to unwanted retinal flow and or efference copy/vestibular signals that might influence judgements. However, we think that (again) the most likely effect would be an increase in noise on settings rather than driving a bias such as that described above. To drive a bias like that observed would presumably require a systematic pattern of eye/head movements in one direction which is unlikely, especially given that participants’ movements were oscillatory in opposing directions and so any unwanted eye/head movement signals should be generated in both directions (and therefore cancel).

A fourth issue with using VR technology (although this is also common for more standard CRT/LCD based vision experiments involving stereoscopic stimuli) is that the effective focus distance (EFD) of the device will drive the accommodative state of the lens, which could lead to conflict between the accommodation and vergence systems (and potentially misestimates of our stimulus distance at 1.5 m) (e.g., see Peillard et al., 2020). If the EFD was actually nearer than 1.5 m, then this would bias the observer to perceive the ball as nearer than 1.5 m, whereas if it were further than 1.5 m, it would lead to the opposite effect. This is relevant since it might contribute to the observed bias via the alternative account described above (involving a misestimate of perceived stimulus distance). Unfortunately, the EFD of the HTC VIVE is not readily available and accurate measurement is not trivial. Several VIVE users have tried to measure this and reported results online, and a survey of these results suggests that EFD is somewhere in the range 0.75–3m. Our stimulus, at 1.5 m, was near the middle of this range, which, in the circumstances, seems like a judicious choice.

### Conclusion

Perceptual stability rests upon our ability to appropriately interpret retinal motion signals, by constantly comparing current retinal input to commensurate information about self-movement from a range of sources. Deviations from perceptual stability arise when these two do not cancel each other out. We highlight here the considerable inaccuracy and imprecision in such compensation processes when key information about self-movement is absent and suggest that this is common across different observer-scene movement scenarios. We suggest that both motor command information (efference copy) and visual information (optic flow) about self-movement are particularly important for this task.

## Supplemental Material

sj-docx-1-pec-10.1177_03010066221116480 - Supplemental material for Investigating distortions in perceptual stability during different self-movements using virtual reality Click here for additional data file. Supplemental material, sj-docx-1-pec-10.1177_03010066221116480 for Investigating distortions in perceptual stability during different self-movements using virtual reality by Paul A. Warren, Graham Bell and Yu Li in Perception
