# Supplementary material for: Investigating distortions in perceptual stability during different self-movements using virtual reality
Source: Perception. 2022 Aug 9;51(10):681–97. doi: 10.1177/03010066221116480 (PMC9478599; doi:10.1177/03010066221116480)
Supplement: sj-docx-1-pec-10.1177_03010066221116480 - Supplemental material for Investigating distortions in perceptual stability during different self-movements using virtual reality [file sj-docx-1-pec-10.1177_03010066221116480.docx]

Supplementary materials for:

Investigating distortions in perceptual stability during self-movements using Virtual Reality

Paul A. Warren*, Graham Bell, Yu Li

Virtual Reality Research (VR2) Facility

Division of Neuroscience and Experimental Psychology

University of Manchester

Oxford Road, Manchester, M13 9PL

*** Corresponding author**

***S1: Derivation of mis-estimated distance travelled***

We begin with the L:L observer-target movement combination as this is the simplest to explain. Figure S1A presents the case where the observer translates a distance $x_{o}$ to the right. When the target has gain $\gamma$ it is displaced to the right through a distance of ${x_{t}=\gamma x}_{o}$. If the bias recovered from the psychometric procedure is $\beta$ then this is the value of $\gamma$ for which the ball is perceived as scene-stationary and accordingly the ball displacement would be ${x_{t}=\beta x}_{o}$ and the relative displacement, $\Delta$, between the observer and target would be:

$\Delta=(1-\beta)x_{o}$. (A1)

The question then is what is the (misestimated) value, $x_{o}^{'}$, of observer displacement for which this same relative displacement between observer and a scene-stationary target would be expected. Clearly from Figure S1A this is $x_{o}^{'}=(1-\beta)x_{o}$ and the ratio of this misestimate to the actual distance moved (the normalised distance) is thus:

$\frac{x_{o}^{'}}{x_{o}}=\frac{(1-\beta)x_{o}}{x_{o}}=(1-\beta)$ (A2)

*Figure S1.1: The biases recovered from the psychometric procedure can potentially be explained as a misestimate of the distance travelled. Here we show how this misestimate,* $x_{o}^{'}$*, relates to other key variables associated* with the observer and target movements.

For the case of S:S a similar logic applies. Figure S1B presents the case where the observer translates a distance $z_{o}$ forwards towards the ball which starts at distance *δ*. When the target has gain $\gamma$ it is displaced in the same direction as the observer through a distance of ${z_{t}=\gamma z}_{o}$. If the bias recovered from the psychometric procedure is $\beta$ then this is the value of $\gamma$ for which the ball is perceived as scene-stationary and accordingly the ball displacement would be ${z_{t}=\beta z}_{o}$ and the relative distance, $\Delta$, between the observer and target would be:

$\Delta= \delta-z_{o}+z_{t} = \delta-(1-\beta)x_{o}$. (A3)

The question then is what is the (misestimated) value, $z_{o}^{'}$, of observer displacement for which this same relative distance between observer and a scene-stationary target would be expected. Clearly from Figure S1A this is given by:

$z_{o}^{'}=\delta-\Delta= (1-\beta)x_{o}$ (A4)

and the ratio of this misestimate to the actual distance moved (the normalised distance) is thus:

$\frac{z_{o}^{'}}{z_{o}}=\frac{(1-\beta)z_{o}}{z_{o}}=(1-\beta)$ (A5)

The case of (L+R):R is slightly more difficult because the observer movement involves both translation and rotation. However, we assume, similar to Tcheang et al., 2005, that the critical variable is the angular offset between the observer’s straight-ahead direction and the direction in which the target faces. Figure S1C shows an observer translating a distance $x_{o}$ while counterrotating the head through angle $\theta$. When the target has gain $\gamma$ it rotates towards the observer about an angle of $\theta_{t}=\gamma\theta$. If the bias recovered from the psychometric procedure is $\beta$ then this is the value of $\gamma$ for which the target is perceived as scene-stationary and accordingly the target rotation would be $\theta_{t}=\beta\theta$ and the angular offset, $\Delta\theta$, between the observer’s straight-ahead direction and the direction in which the target faces would be:

$\Delta\theta=(1-\beta)\theta$ (A4)

The question now is what is the (misestimated) value, $x_{o}^{'}$, of observer displacement for which this same relative angular offset between observer and a scene-stationary target would be expected. Under the assumption that perceived distance to the target, $\delta$, is approximately correct then from Figure S1C we know that $x_{o}^{'}=\delta\tan\Delta\theta$ and similarly $x_{0}=\delta\tan\theta$, so the normalised distance moved is then given by:

$\frac{x_{o}^{'}}{x_{o}}=\frac{\tan\Delta\theta}{\tan\theta}=\frac{\tan(\left( 1-\beta\right)\theta)}{\tan\theta}$ (A5)

For small angles (A5) is approximated by $(1-\beta)$ and for consistency with the other observer-target movement combinations outlined above, this value is plotted in Figure 4.

***S2: Descriptive statistics for PSS and Gaussian s.d. parameters***

*Table S2.1: means and 95% confidence intervals for fitted PSS parameter (bias)*

|  | (L+R):R | S:S | L:L |
| --- | --- | --- | --- |
| Active  *m*  95% CI-  95% CI+ | 0.173  0.051  0.294 | 0.148  0.038  0.257 | 0.220  0.116  0.323 |
| Passive  *m*  95% CI-  95% CI+ | 0.286  0.165  0.406 | 0.278  0.153  0.403 | 0.318  0.198  0.439 |

*Table S2.2: means and 95% confidence intervals for fitted Gaussian s.d. parameter*

|  | (L+R):R | S:S | L:L |
| --- | --- | --- | --- |
| Active  *m*  95% CI-  95% CI+ | 0.304  0.198  0.410 | 0.239  0.185  0.293 | 0.221  0.167  0.274 |
| Passive  *m*  95% CI-  95% CI+ | 0.378  0.221  0.535 | 0.319  0.255  0.384 | 0.263  0.206  0.321 |
